# Effectiveness of the Mobility Rehab System for Mobility Training in Older Adults: A Pragmatic Clinical Trial

**DOI:** 10.3389/fneur.2021.680637

**Published:** 2021-09-06

**Authors:** Rodrigo Vitório, Mahmoud El-Gohary, Sean Pearson, Patricia Carlson-Kuhta, Graham Harker, Fay B. Horak, Jodi Lapidus, Mike Studer, Martina Mancini

**Affiliations:** ^1^Department of Neurology, Oregon Health and Science University, Portland, OR, United States; ^2^APDM Wearable Technologies – an ERT Company, Portland, OR, United States; ^3^Biostatistics and Design Program Core, Oregon Health and Science University, Portland, OR, United States; ^4^Northwest Rehabilitation Associates, Salem, OR, United States

**Keywords:** rehabilitation, biofeedback, gait, balance, mobility

## Abstract

**Introduction:** Mobility impairments are among the main causes of falls in older adults and patients with neurological diseases, leading to functional dependence and substantial health care costs. Feedback-based interventions applied in controlled, laboratory environments have shown promising results for mobility rehabilitation, enhancing the benefits of standard therapy. However, the effectiveness of sensor-based feedback to improve gait in actual outpatient physical therapy settings is unknown. The proposed trial examines the effectiveness of a physical therapist-assisted, visual feedback system using wearable inertial sensors, Mobility Rehab, for mobility training in older adults with gait disturbances in an outpatient clinic.

**Methods:** The study is a single site, pragmatic clinical trial in older adults with gait disturbances. Two hundred patients undergoing their outpatient rehabilitation program are assigned, by an independent assistant, for screening by one of four therapists, and assigned to either a standard physical therapy or therapist-assisted feedback therapy. Both groups train twice a week for 6 weeks. Four physical therapists were randomized and stratified by years of experience to deliver standard therapy or therapist-assisted feedback rehabilitation. Each session is 45 min long. Gait is trained for 30 min. The additional 15 min include exercises for endurance, strength, and static and dynamic balance in functional tasks. Mobility Rehab uses unobtrusive, inertial sensors on the feet and belt with real-time algorithms to provide real-time feedback on gait metrics (i.e., gait speed, double support time, foot clearance, angle at foot strike, and arm swing), which are displayed on a hand-held monitor. Blinded assessments are carried out before and after the intervention. The primary outcome measure is subjects' perception of balance as measured by the Activities-specific Balance Confidence scale. Gait speed, as measured with wearable inertial sensors during walking, is the secondary outcome measure.

**Discussion:** We hypothesize that therapist-assisted feedback rehabilitation will be more effective than standard rehabilitation for gait. Feedback of motor performance plays a crucial role in rehabilitation and objective characterization of gait impairments by Mobility Rehab has the potential to improve the accuracy of patient-specific gait feedback.

**Clinical Trial Registration:**www.ClinicalTrials.gov, identifier: NCT03869879.

## Introduction

Gait training in physical therapy has been shown to improve gait (1–4). However, benefits are not optimal yet. Often, it is difficult for physical therapists (PTs) to observe subtle abnormalities in gait patterns and difficult to provide quick accurate feedback about gait impairments to their patients. For example, abnormalities of duration of double support time, foot clearance or angle of the foot at heel strike may be difficult to observe, yet they are important characteristics to improve to avoid falls (5, 6). PTs and patients would benefit from using a reliable system to efficiently and accurately quantify gait impairments, provide cues on how to modify abnormal gait patterns and quantify each patient's response to therapy.

Gait and balance impairments are among the most common causes of falls in older adults and neurologic patients (7, 8). Gait impairments often manifest as slow gait with increased double support time, reduced foot clearance, reduced angle at foot strike, decreased arm swing, and increased variability of these measures (5, 9, 10). These impairments are usually multifactorial (11) and require a comprehensive assessment to identify persons at increased risk of falling and inform interventions (5). Many falls could be preventable through improved screening for gait disorders and training of those at increased risk (12, 13).

Feedback-based systems have been successfully used in laboratories for the treatment of gait abnormalities in clinical populations (14–17), exceeding the efficacy of standard physical therapy (18). However, most gait feedback systems are limited to treadmill walking, which is different from over-ground walking (19, 20), and they are limited to lower limb-related metrics. Mobility Rehab (APDM Wearable Technologies – an ERT company, Portland, Oregon, USA) was developed for both overground and treadmill walking and provides feedback about trunk and upper limb movements, as well as lower limb movements. The system uses wireless, wearable, inertial sensors (Opals) on the wrists, feet and trunk to improve the accuracy and effectiveness of PTs' feedback to their patients by providing objective measures of gait in real-time. Mobility Rehab provides therapists stride-by-stride, relative measures of gait quality, as well as summary statistics, on a tablet as their patient walks over-ground in natural conditions so they can provide quick, accurate verbal instructions to their patients.

There is a need to establish the effectiveness of therapist-assisted feedback in an actual outpatient therapy setting. The effect of feedback-assisted rehabilitation of gait and balance has mostly been investigated in laboratory settings (18, 21). It is unknown if successful application of feedback-based interventions is effective in an actual outpatient physical therapy clinic that provides gait and balance training to older adults with mobility disturbances. Here, we will conduct a pragmatic clinical trial in a large outpatient clinic to examine the effectiveness of the Mobility Rehab system for mobility training in older adults with gait disturbances. We hypothesize that therapist-assisted feedback rehabilitation will be more effective than standard rehabilitation for gait.

## Methods and Analysis

### Trial Design

**Figure 1:**
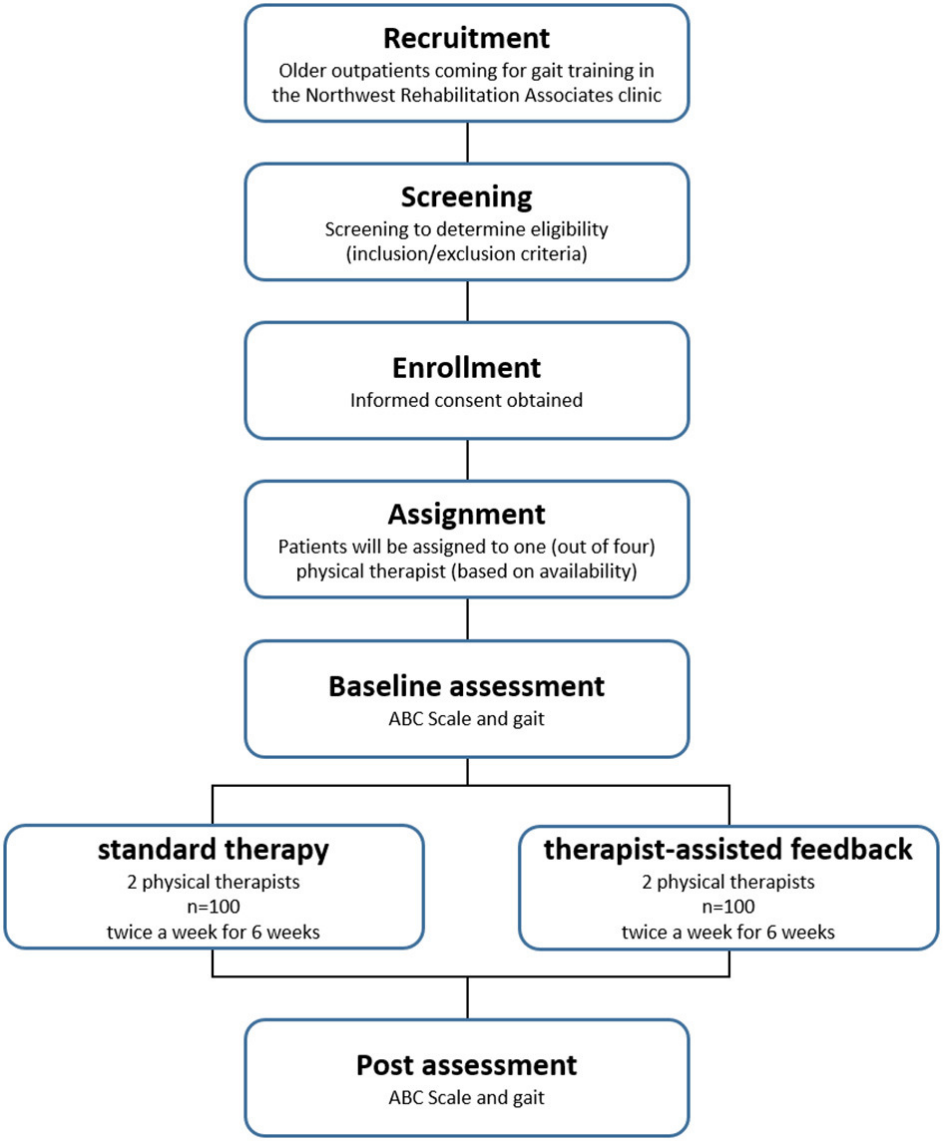
Flowchart of the study.

**Figure 2:**
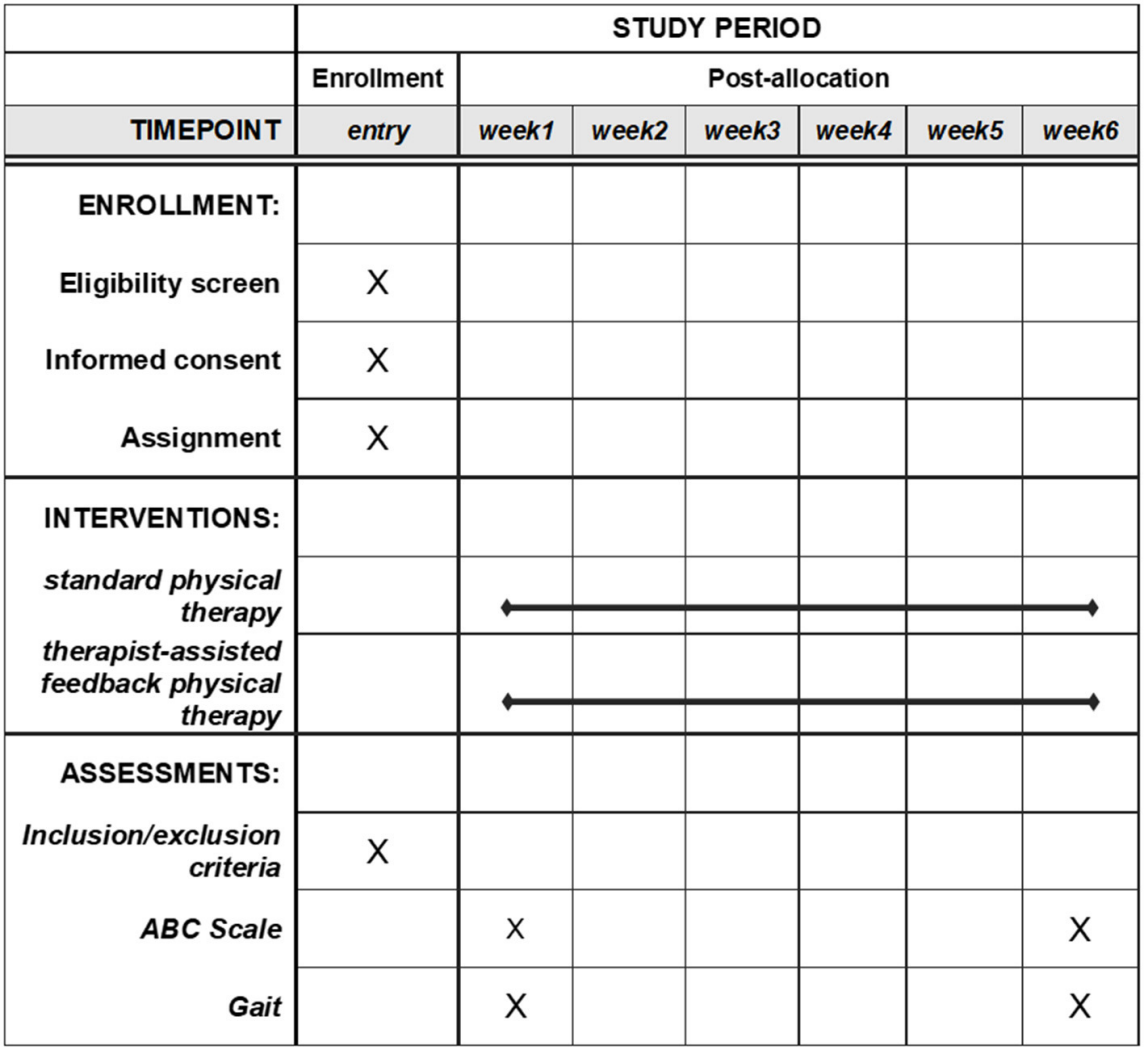
SPIRIT template for the schedule of enrollment, interventions, and assessments.

The study is a single-site, pragmatic, controlled clinical trial in older people with gait disturbances. The trial compares standard-of-care gait training by PTs with therapist-assisted feedback therapy using Mobility Rehab in the same clinic. Participants scheduled at the physical therapy clinic for gait training are assigned to one of the four PTs, and that will determine, if eligible, their group assignment for the duration of their outpatient therapy. Assessments occur at baseline (pre) before intervention starts and immediately after the last session of intervention (post). The trial design is illustrated in Figure 1. This protocol paper follows the SPIRIT (Standard Protocol Items: Recommendations for Interventional Trials) 2013 statement and guidelines (22). SPIRIT template for the schedule of enrollment, interventions, and assessments is presented in Figure 2.

### Study Setting

The intervention sessions and blinded assessments are performed at Northwest Rehabilitation Associates, an outpatient rehabilitation center in Salem (Oregon, USA). The clinical trial outcomes will be analyzed and summarized by blinded investigators and statisticians from Oregon Health & Science University (OHSU, Portland, Oregon, USA).

### Recruitment and Screening

Outpatients coming for gait training at the Northwest Rehabilitation Associates clinic are invited to participate in this trial. Those who agree to learn more about the study are seen by a PT aide who explains the study further, completes a screening interview (for exclusion and inclusion), supervised by an experienced PT (MS), and obtains consent. There are no gender, ethnic, or racial minority exclusions for this study. Inclusion criteria include: 60–89 years old and gait disturbances requiring physical therapy. Any disease may be included and efforts are made to maintain an equal number of women and men in the sample. Participants are included in the study if they are able to follow instructions (up to the PT's judgement). This trial will stop enrolling participants when 200 individuals will have completed baseline and follow-up assessment.

### Assignment and Blinding

A PT aide who schedules patient appointments assigns each prescreened patient to one of the four PTs who deliver the interventions, depending their open schedule. If a patient is eligible, then he/she undergoes the intervention with the assigned PT. Thus, although the patients are not randomly assigned to each group, this approach was practical for the clinic and avoided selective enrollment assignment since the PT aide assigns an upcoming patient to a PT according to schedule availability without regard to diagnosis, age, or sex. To prevent performance bias, the four treating PTs were distributed into two groups: two PTs provide traditional physical therapy for 100 participants, while the two other PTs provide feedback-based therapy for the other 100 participants. We do not anticipate any selection bias and we do expect equal number of subjects to be assigned to each of the four PTs. Furthermore, information that could introduce bias are withheld from subjects in both groups, i.e., their informed consent does not mention efficacy of feedback-based therapy. To prevent bias due to varying levels of PTs experience, one very experienced PT (>10 years working with patients) and one less experienced PT (<5 years working experience) were assigned to use Mobility Rehab and a similar stratification of experienced and inexperienced PTs were assigned to not use Mobility Rehab. Detection bias is avoided by blinding the PT assistants who administer the pre- and post-assessments and by blinding the investigators and statisticians at OHSU who are analyzing the results regarding subjects' group assignment.

Although we are not assessing blinding by the outcome assessors, the following measures are implemented to avoid assessors from gaining impressions/information about treatment assignment: assessors are based in a different building from the one where the interventions are carried out and, therefore, they do not see patients during rehabilitation sessions and; participants are asked to refrain from mentioning their group allocation to assessors.

### Interventions

The two rehabilitation interventions for gait disturbances are: (1) standard-of-care physical therapy and (2) therapist-assisted feedback physical therapy. Both groups train twice a week for 6 weeks. Sessions are 45 min long and gait (with or without Mobility Rehab) is trained for 30 min in each session. The additional 15 min include exercises for endurance, strength, and static and dynamic balance in functional tasks. The difference between the two groups is the use of the Mobility Rehab system in the therapist-assisted feedback group. PTs design their own treatment plan for each patient. They are allowed to select modality, overground walking and/or treadmill, and tasks (dual task, head turns, etc.) as appropriate for each patient. During a regular session, patients work on gait with the following tasks for 30 min: weights on ankles, dual tasks, upper extremity support, partial body weight support, speed challenges, direction changes, obstacles, and head turning. The PTs using Mobility Rehab system underwent specific training on how to effectively use the system (e.g., sensors placement, software navigation, selection and interpretation of metrics, etc.) before the start of the trial.

**Figure 3:**
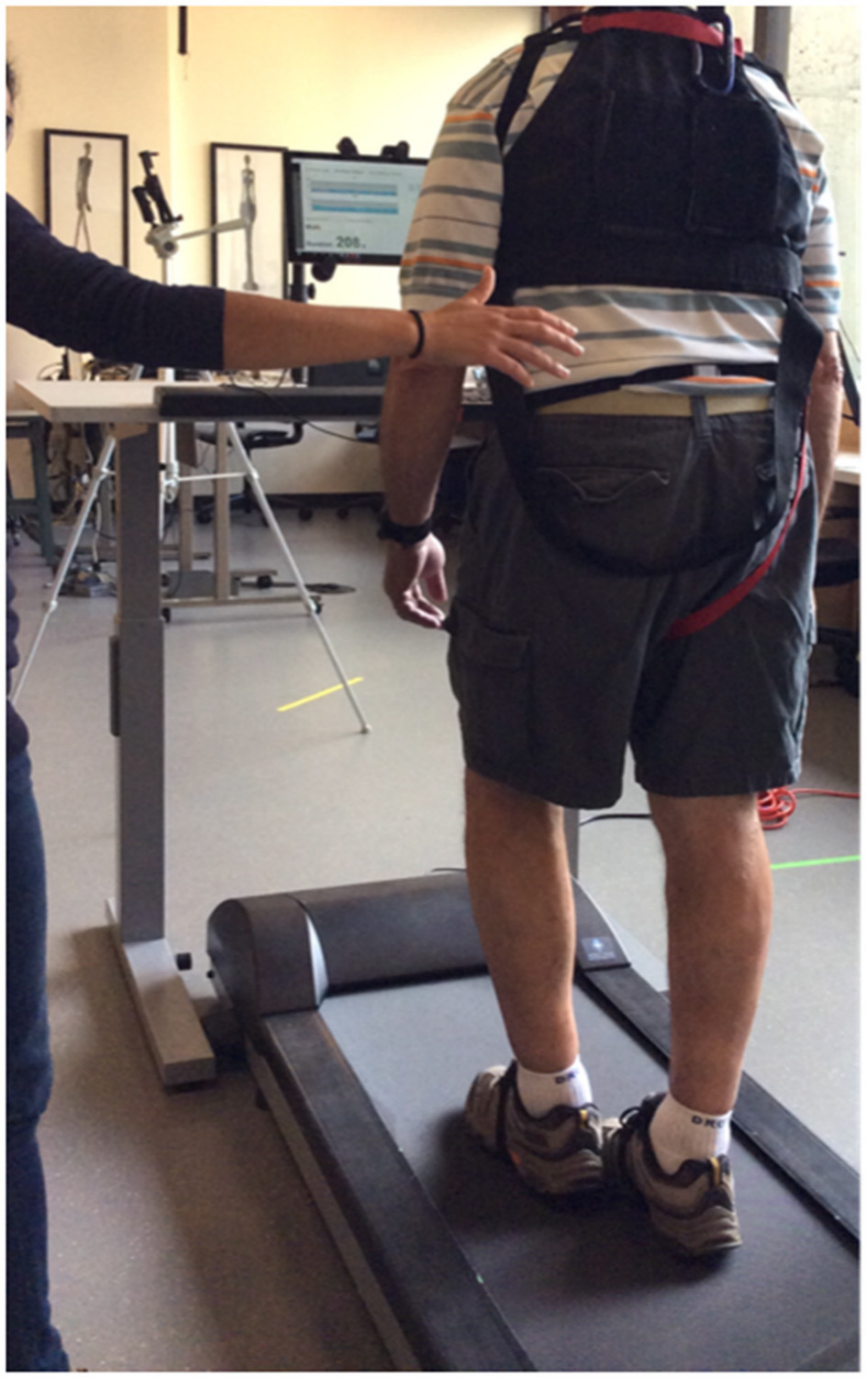
Photograph of a patient using Mobility Rehab with a PT during a therapy session.

The Mobility Rehab system uses unobtrusive, wearable, inertial sensors with real-time algorithms to provide real-time feedback on five gait metrics: step duration, stride length, arm range of motion, trunk coronal range of motion, and elevation at midswing (Figure 3). Therapists are allowed to select any gait metric and to focus on one or several gait metrics. The visual feedback is provided to a hand-held monitor/tablet (Figure 3) and displayed relative to age-specific normative values, which were previously collected from 120 healthy subjects between the ages of 60 and 89 years during a 2-min overground walk at a comfortable pace. Therapists also can select a minimum and maximum goal for a metric, based on each patient's abilities.

**Figure 4:**
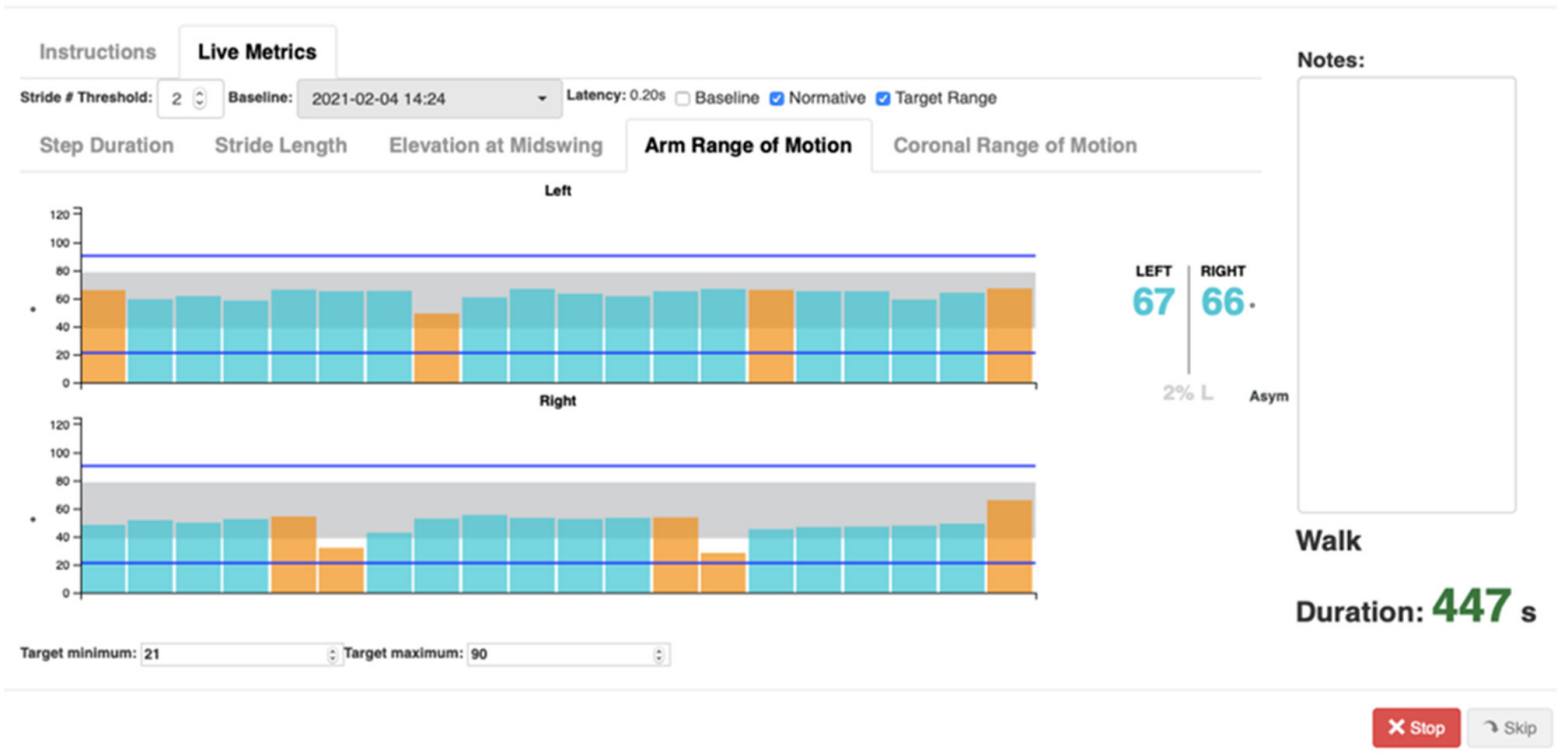
Mobility Rehab interface and visualization of gait metrics while walking. Example of biofeedback visualization of arm range of motion while walking. Bars indicate left and right arm range of motion during straight walking (blue) and 180° turns (orange).

The Mobility Rehab system includes a tablet (to visualize gait measures) and five Opal sensors (APDM, Portland, Oregon, USA), that are placed on both wrists and feet and at the sternum level. The wireless sensors are easy to attach with Velcro straps and highly portable, enabling assessment to be performed in almost any environment. Figure 4 shows a screen-shot of the display.

The user can see metrics for both the right and left sides (top panel and bottom panel) in which each bar represents the value of one step/stride and the gray area represents the normative values. In addition, intervals can be set as target (blue lines) to reach while walking. Each time a new step/stride is added, a new bar is appended and appears on the graph. The system will record each metric used for each session, length of the session, and if the training was on treadmill or overground. In addition, the system provides therapists and patients with online reports that convey patients' performance during the training session and a comparison to baseline gait data.

### Adverse Events

Adverse events during the intervention period are reported to the research group by the training therapists. The investigators report adverse events and unanticipated problems in accord with the policies of the OHSU Institutional Review Board. If trends in adverse events are noted, preventive measures will be implemented and participants will receive advice on avoiding such events.

### Outcomes and Procedures

The primary outcome measure is the subjects' perception of balance confidence as the patient-related-outcome (common in pragmatic trials). Subjects' perception of balance is measured with the Activities-specific Balance Confidence scale (ABC). The ABC consists of 16 questions about subjects' confidence in not losing balance while engaged in daily-life activities, such as picking something up from the floor, riding an escalator, and walking across a parking lot. Subjects respond on a scale from 0 to 100%, representing the confidence they have in their balance when doing or imagine if they did these tasks. ABC score above 50 and lower than 80 indicates a moderate level of functioning characteristics of elders in retirement homes and persons with chronic health conditions (23). The ABC has been shown to reflect the activities in which people actually participate that involve walking (24).

The secondary outcome measure is gait speed, as it has been reported as the 6th vital sign and predictive of mortality (25). Gait speed is measured during two instrumented, 2-min walks. Participants are invited to walk back and forth along a 9-meter long corridor. The first walk bout is performed at the individual's casual walking pace and the second is performed at a pace the individual considers to be faster than their casual pace. The Mobility Lab software is used to collect the walking data and calculate gait speed and additional spatiotemporal gait parameters (i.e., step time asymmetry, foot clearance, arm swing, trunk lateral range, and foot strike angle), which will be reported as exploratory outcomes in this trial (26).

### Sample Size and Power Analysis

#### ABC Scale

We anticipate most of our subjects will score in the 50–80 range on the ABC scale at baseline, although we will not exclude those with scores lower than 50. We extracted means and standard deviations (SDs) from published studies involving both community dwelling and home-care facility based older adults (23). Based on these studies, we assume our subjects will have an average score of 60 at baseline with a SD in the 11–20 range. Furthermore, a 12-point change in the scale has been reported to be clinically relevant (27). We anticipate that both the standard therapy and therapist-assisted feedback treatment groups will improve, but the feedback group will score at least 12 points higher on ABC scale at post-assessment. Thus, we anticipate group effect sizes between 0.6 and 1.2 SD for the current study. Type 1 error for the sole primary outcome was set to 0.05. Because we propose to randomize PTs rather than patients, there is clustering inherent in the study design (i.e., cluster-randomization). We account for clustering by computing power based on the effective sample size rather than the actual sample size (28). The effective sample size (ESS = m ^*^ (number of therapists)/DEFF) is based on the design effect (DEFF = 1 + (m – 1) ^*^ ICC), where ICC = intra-cluster (therapist) correlation and m = 50 is the anticipated # patients/therapist. We examined power to detect anticipated changes for a range of ICC between 0.002 and 0.010 [are based on values reported by Killip et al. (28)]. With 200 subjects equally allocated to each of the four therapists (two of which are randomized to therapist-assisted feedback group), the trial is adequately powered (84.3–96.1%) to detect effect sizes between 0.9 and 1.2 SD when ICC = 0,002; effect size of 1.1 SD is detectable with 82.6–91.1% power when ICC is higher (0.10).

#### Gait Speed

Under similar assumptions for ICC, we computed power to detect changes assuming a sample size of 200 allocated as noted above. Evaluated group differences for gait speed between 0.06 and 0.12 (29). Since gait speed is not the primary outcome, we did not control for multiple comparisons, and left type 1 error equal to 0.05. Power is ≥85.7% to detect at least 0.12 m/s difference when ICC is at or below 0.005, and ≥80.5% to detect at least 0.12 m/s difference when ICC is 0.01.

### Statistical Analysis

The analysis will adopt the intent-to-treat strategy. We will estimate and evaluate the difference in the post-assessment ABC between the two treatment groups, adjusting for participants' baseline characteristics (i.e., age, gender, and pathology). Since the outcome of interest is measured at the individual patient level, but PTs are the unit of randomization (i.e., PTs are assigned to either provide feedback in addition to usual care or not), we will fit a linear mixed effects model with post-assessment ABC as the dependent variable; fixed independent effects will be treatment group (therapist-assisted feedback therapy vs. standard therapy) and a random effect term to account for PT variability. The same model will be used to investigate changes on gait speed, our secondary outcome measure. Additionally, we will carry out a sensitivity analysis for those that complete at least 9 (out of 12) sessions to identify whether or not a certain number of sessions influences the observed effects, if any.

An exploratory analysis is proposed including estimating post-intervention differences in other gait metrics collected by the blinded assessor. These secondary gait outcomes will be fit with models similar to the primary outcome measure.

## Discussion

This study will determine whether Mobility Rehab is effective for mobility training in older adults with gait disturbances in an outpatient physical therapy clinic setting. We hypothesize that therapist-assisted feedback rehabilitation using Mobility Rehab will be more effective than standard rehabilitation for gait because sensory feedback of performance plays a crucial role in motor rehabilitation. Currently, PTs observe patients' overall walking patterns and provide occasional verbal and/or somatosensory feedback to improve their patients' gait. Although the current approach allows patients to become more aware of how they move so they can correct any abnormal gait strategies, these methods are not optimal. In fact, gait observation is subjective and might be inaccurate, especially for gait impairments that are difficult to observe, such as an excessive trunk motion or reduced foot clearance. An objective characterization of gait impairments is therefore required for accurate patient-specific gait feedback, which may lead to optimized benefits. Additionally, the broad range of patient characteristics (age, gender, diagnosis, mobility condition) in this study will enable the results to be extrapolated to the whole population of elderly patients in need of gait training.

## Ethics Statement

Ethics approval was obtained from the Institutional Review Board at OHSU (eIRB # 16282). Written informed consent will be obtained from each participant prior to undertaking any trial activities.

## Author Contributions

RV drafted the manuscript. ME-G, MS, FBH, JL, and MM designed the study and edited the manuscript. GH and MS were coordinating the study and edited the manuscript. MS provided expertise on inclusion and exclusion criteria. SP and PC-K revised the manuscript. All authors read and approved the final manuscript.

## Author Disclaimer

The content is solely the responsibility of the authors and does not necessarily represent the official views of the National Institutes of Health.

## Conflict of Interest

FBH and ME-G are employees of APDM Wearable Technologies-an ERT company, a company that may have a commercial interest in the results of this research and technology. This potential conflict has been reviewed and managed by OHSU. In addition, SP was employed by APDM Wearable Technologies-an ERT company. FBH also consults with Autobahn, Biogen, Pfizer, Medtronic, Neuropore, Sanofi, and Takeda. The remaining authors declare that the research was conducted in absence of any commercial or financial relationship that could be construed as a potential conflict of interest.

## Publisher's Note

All claims expressed in this article are solely those of the authors and do not necessarily represent those of their affiliated organizations, or those of the publisher, the editors and the reviewers. Any product that may be evaluated in this article, or claim that may be made by its manufacturer, is not guaranteed or endorsed by the publisher.
